# Expectancies as predictors of symptom improvement after antimicrobial therapy for persistent symptoms attributed to Lyme disease

**DOI:** 10.1007/s10067-021-05760-1

**Published:** 2021-05-24

**Authors:** Henriët van Middendorp, Anneleen Berende, Fidel J. Vos, Hadewych H. M. ter Hofstede, Bart Jan Kullberg, Andrea W. M. Evers

**Affiliations:** 1 Health, Medical and Neuropsychology Unit, Faculty of Social and Behavioural Sciences, Institute of Psychology, Leiden University, PO Box 9555, 2300 RB Leiden, The Netherlands; 2 Department of Medicine, Radboud Center for Infectious Diseases, Radboud University Medical Center, Nijmegen, The Netherlands; 3 Gelre Ziekenhuizen, Apeldoorn, The Netherlands; 4 Sint Maartenskliniek, Nijmegen, The Netherlands; 5 Department of Psychiatry, Leiden University Medical Center, Leiden, The Netherlands

**Keywords:** Antibiotics treatment, Expectancies, Lyme disease, Psychology, Treatment outcome

## Abstract

**Introduction/Objective:**

Expectancies about symptom improvement or deterioration are reliable predictors of symptom progression and treatment outcomes (symptom resolution or symptomatic improvement) in many (non-)pharmacological studies and treatments. This study examined predictors of symptom improvement after antimicrobial therapy for persistent symptoms attributed to Lyme disease, hypothesizing particularly pre-treatment expectancies regarding symptom improvement to be predictive.

**Methods:**

A predictive study was performed on pre-treatment and post-treatment individual characteristics, including expectancies, and physical and mental health–related quality of life (HRQoL) from the PLEASE-trial comparing randomized 12-weeks of doxycycline, clarithromycin-hydroxychloroquine, or placebo following 2 weeks of intravenous ceftriaxone. At end-of-treatment (14 weeks after trial start) and follow-up (52 weeks), complete data of 231 and 170 (of initial 280) patients with persistent symptoms temporally related to a history of erythema migrans or otherwise confirmed symptomatic Lyme disease, or accompanied by *B. burgdorferi* IgG or IgM antibodies, were examined through hierarchical regression analyses.

**Results:**

In addition to pre-treatment HRQoL, pre-treatment expectancies regarding symptom improvement were consistently associated with stronger physical and mental HRQoL improvements at both end-of-treatment and follow-up (95% CI range: .09;.54, *p* < .01 to .27;.92, *p* < .001). Post-treatment expectancies regarding having received antibiotics vs. placebo was associated with more HRQoL improvement at end-of-treatment, but not at follow-up (95% CI-range 1.00;4.75, *p* = .003 to −7.34; −2.22, *p* < .001).

**Conclusions:**

The present study shows that, next to pre-treatment functioning, patients’ pre-treatment and post-treatment expectancies regarding improvement of persistent symptoms attributed to Lyme disease relate to a more beneficial symptom course. Expectancies of patients may be relevant to explain and potentially improve patient outcomes (e.g., by optimized communication about treatment success).

**Trial registration:**

ClinicalTrials.gov, NCT01207739 (Registration date: 23–09-2010)

**Table:** 

**Key Points** • *As there is currently no sufficient symptom resolution or symptomatic improvement for many patients with persistent symptoms attributed to Lyme disease, it is relevant to know which factors determine symptom progression and predict heterogeneity in treatment response*. • *Next to pre-treatment functioning, expectancies regarding symptom improvement and having received antimicrobial study medication are associated with a more beneficial symptom course after both shorter-term and longer-term antimicrobial treatment.* • *Expectancies are relevant to consider in treatment studies and may be useful in clinical settings to improve symptom course and treatment outcome (e.g., by optimized communication about treatment success).*

## Introduction

Large numbers of patients present with persistent symptoms attributed to infection with *Borrelia burgdorferi* [1, 2]. These patients mainly experience disabling symptoms of pain, fatigue, and neurological and cognitive disturbances. The most commonly provided current medical treatment consists of either shorter-term (2–4 weeks) or longer-term (≥ 3 months) antimicrobial therapy. Previous studies have indicated that, despite positive outcomes for a subset of patients, current standardized protocols of shorter-term and longer-term antimicrobial therapies do not offer a sufficient symptom resolution or symptomatic improvement for many patients with persistent symptoms [3–6]. Thus, it is relevant to know which factors determine symptom progression and predict heterogeneity in treatment response, as this could offer potential new ways to individualize treatment and improve patient outcomes for a larger patient group.

Various demographic, disease-related, and individual characteristics, as well as pre-treatment functioning, have been related to treatment outcomes (symptom resolution or symptomatic improvement) in previous studies in diverse populations, including patients with persistent symptoms attributed to Lyme disease. However, the results were inconsistent, limiting the clinical implications of these findings [7–13]. More recently, particular interest has been devoted to the role of expectancies of patients regarding their symptom progression and treatment outcomes. In many placebo-controlled trials, expectancies are reliable predictors of treatment outcomes in a broad variety of pharmacological and non-pharmacological treatments [14–20]. Moreover, some studies have shown that the drug that patients thought they received (active or placebo) was more strongly related to outcome than the actual drug received [21, 22]. As increasingly acknowledged and starting to be applied in clinical populations [23–28], expectancies may thus strengthen or even partly determine the effects of these treatments.

The current study examines the role of pre-treatment and post-treatment expectancies in comparison to other individual characteristics in predicting primary outcomes of symptom improvement after antimicrobial therapy for persistent symptoms attributed to Lyme disease. We hypothesized that, in addition to pre-treatment functioning, particularly pre-treatment expectancies regarding symptom improvement would be predictive of changes in physical and mental health-related quality of life (HRQoL) immediately after treatment and at the longer term.

## Materials and methods

### Participants and procedure

The current study concerns a secondary analysis of the data collected as part of the Persistent Lyme Empiric Antibiotic Study Europe (PLEASE) [3], a multicentre, placebo-controlled, double-blind randomized controlled trial conducted at two tertiary health centers in the Netherlands, the Radboud University Medical Center and the Sint Maartenskliniek. The PLEASE study examined whether two specific standardized longer-term antimicrobial therapies would lead to better patient outcomes than a 2-week standardized shorter-term antimicrobial therapy followed by placebo in patients with symptoms attributed to Lyme disease. The trial was registered at ClinicalTrials.gov (NCT01207739) and its design and main results have been reported in previous papers [3, 29]. In short, 280 patients with persistent symptoms (e.g., pain, musculoskeletal symptoms, neuralgia, sensory disturbances, neuropsychological complaints, fatigue) that were either temporally related to a history of an erythema migrans (EM) or otherwise confirmed symptomatic Lyme disease, or accompanied by *B. burgdorferi* IgG or IgM antibodies were included into the trial. All participants received 2000-mg open-label intravenous ceftriaxone daily for 2 weeks (shorter-term treatment) before starting a blinded oral antibiotic regimen of 12 weeks (longer-term treatment), for which they were randomly allocated in a 1:1:1 ratio to one of three treatment arms: (1) 100 mg of doxycycline twice daily plus placebo twice daily; (2) 500 mg clarithromycin twice daily plus 200 mg hydroxychoroquine twice daily; or (3) two placebos twice daily. The data for the current study were derived from questionnaires assessed at study start, at 14 weeks (end-of-treatment; primary outcome assessment point) and 52 weeks (long-term follow-up) after study start. The study was ethically approved by the Medical Ethics Review Committee CMO Region Arnhem-Nijmegen, and all participants gave written informed consent.

## Instruments

### Primary and secondary outcome measures

The same primary and secondary physical and mental health–related quality of life (HRQoL) outcome measures as in the PLEASE trial [3] were examined in this study.

#### Physical HRQoL

##### Physical component summary score

As primary outcome, the physical component summary score (PCS) of the RAND-36 Health Status Inventory (RAND SF-36) was assessed [30]. This score is calculated as norm-based *T*-score from the four weighted physical subscales (physical functioning, role limitations due to physical health problems, pain, and general health perceptions); these *T*-scores range from 15 to 61 and have a mean of 50 and standard deviation of 10 in the general population, with higher scores indicating a better physical HRQoL.

##### Fatigue severity

Fatigue, which is a frequent symptom that is not measured within the PCS of the RAND SF-36, was assessed as a secondary outcome by means of the severity of fatigue subscale of the Checklist Individual Strength (CIS) [31]. This is an 8-item scale with a score range of 8 to 56, with a mean of 17 and a standard deviation of 10 in a healthy sample. Higher scores on this measure indicate more severe fatigue.

#### Mental HRQoL

##### Mental component summary score

As another secondary outcome, the mental component summary score (MCS) of the RAND SF-36 [30] was assessed, calculated as norm-based *T*-scores (*M* = 50, SD = 10 in the general population) from the four weighted mental subscales (emotional well-being, role limitations due to emotional problems, social functioning, and energy). The *T*-scores on this measure range from 11 to 66, with higher scores indicating a better mental HRQoL.

### Predictors of symptom improvement after antimicrobial therapy

Three categories of predictor variables were assessed: (1) demographic, disease-related, and study-related characteristics; (2) pre-treatment functioning; and (3) individual characteristics, including expectancies.

#### Demographic, disease-related, and study-related characteristics

The following demographic characteristics were assessed pre-treatment: age, sex, marital status, education level, smoking, and paid labor. Disease-related factors assessed pre-treatment were duration of symptoms attributed to Lyme disease and use of pain medication at start of study. The study-related factor included was the randomized treatment arm (doxycycline, clarithromycin plus hydroxychloroquine, or placebo).

#### Pre-treatment functioning

Pre-treatment scores on the primary and secondary outcome measures of physical and mental HRQoL were assessed before randomization and trial-treatment.

#### Individual characteristics

The following variables were assessed at pre-treatment as possible predictors of the treatment outcome:

##### Pre-treatment expectancies regarding symptom improvement

To evaluate expectancies on symptom progression, six items assessed the degree to which participants expected that their symptoms would disappear in the upcoming period (e.g., “I think that my complaints will totally disappear during the upcoming 6 months” and “I think that I will no longer need any medical help for my complaints in the future”), in line with previous studies [18, 32]. Items could be answered on a 4-point Likert scale, varying from 1 “largely disagreed” to 4 “largely agreed,” with a sum score between 6 and 24. A higher score indicates higher expectancies of improvement regarding the course of symptoms. The internal consistency (Cronbach’s alpha = 0.88) was good.

##### Self-efficacy

To determine self-efficacy, six statements on arthritis self-efficacy [33] were adapted to a Lyme Self-Efficacy (LSE) scale, in which the word “pain” was replaced with “physical symptoms” (e.g., “I am certain that I can control my physical symptoms”). Items are answered on a 1 (“totally disagree”) to 5 (“totally agree”) Likert scale, summing up to a score between 6 and 30, with higher scores indicating more self-efficacy. Cronbach’s alpha was 0.78.

##### Illness cognitions

The illness cognitions of helplessness (e.g., “Because of my illness I miss the things I like to do most”), acceptance (“I have learned to accept the limitations imposed by my illness”), and perceived benefits (“Dealing with my illness has made me a stronger person”) were assessed by means of three 6-item scales of the Illness Cognition Questionnaire (ICQ) [34]. Items are answered on a 1 (“not at all”) to 4 (“completely”) Likert scale, adding up to a score between 6 and 24, with higher scores indicating higher levels of helplessness, acceptance, or perceived benefits, respectively. Cronbach’s alphas were 0.87 for helplessness and acceptance, and 0.84 for perceived benefits.

##### Worrying

To assess worrying, the Penn-State Worry Questionnaire (PSWQ) [35]) was used, including 14 statements (e.g., “I know I shouldn’t worry about things, but I just can’t help it”) measuring the tendency, intensity, and uncontrollability of worrying on a scale of 1 (“not at all typical of me”) to 5 (“very typical of me”). Higher scores indicate more worrying. Cronbach’s alpha was 0.93.

##### Personality

By means of the Eysenck Personality Questionnaire [36], neuroticism (22 items, e.g., “Does your mood often go up and down?”) and extraversion (19 items, e.g., “Do you enjoy meeting new people?”) were assessed by means of yes/no answers. Higher scores indicate more neuroticism and extraversion, and Cronbach’s alphas were 0.87 and 0.86, respectively.

Post-treatment, one additional expectancy variable was assessed:

##### Post-treatment expectancies of presumed study medication

At the end-of-treatment (week 14), when returning the study medication bottles, participants were asked what medication they thought they had received, with answering options “antibiotics,” “placebo,” or “do not know.” To make this factor analyzable in regression equations, the variable was converted into “antibiotics” (score 1) or “placebo/don’t know” (score 0).

### Data analysis

To have comparable analyses across outcome measures per assessment point, analyses were performed on complete data sets of all predictor and outcome measures, leading to a sample of 231 patients at end-of-treatment and 170 at follow-up. Descriptive statistics were computed, and changes in HRQoL between the different assessment points were assessed by means of paired-samples *t*-tests. To determine which factors could potentially impact quality of life change, zero-order associations of demographic, disease-related, study-related, and individual characteristics with the outcome measures at week 14 and 52, controlled for pre-treatment HRQoL, were examined by means of analyses of covariance (categorical characteristics) or partial correlations (continuous characteristics). Sex, age, use of pain medication, and acceptance (of the ICQ) did not show significant zero-order associations with any of the quality of life changes in outcome measures at end-of-treatment or follow-up. This held both in the total group and in separate analyses for the two combined longer-term treatment arms and the shorter-term followed by placebo treatment arm. Also, in line with the main results of the PLEASE-trial [3], the treatment arm was not associated with any of the quality of life changes in outcome measures, also when the two longer-term treatment arms were combined (all *p*-values ≥ 0.37). Therefore, these variables were not included in the regression analyses, with the main analyses being conducted in the total group, followed by sensitivity analyses for the shorter-term treatment arm and the combined longer-term treatment arms.

To examine the relative contribution of expectancies and other individual characteristics on physical and mental HRQoL after antimicrobial therapy, separate hierarchical regression analyses were conducted per outcome measure (PCS, fatigue, MCS). In the first block, demographic, disease-related, and study-related characteristics were included. In the second block, the pre-treatment score of the outcome measure was included to control for cross-sectional variance with the other predictor variables, enabling the prediction of changes in HRQoL from pre-treatment to end-of-treatment or follow-up. In the third block, individual characteristics were entered. In order to ensure the most parsimonious model testing, definitive model testing was performed with only those predictor variables that showed at least one significant predictive association across all regression analyses.

To examine whether post-treatment expectancies regarding presumed study medication were related more strongly to HRQoL improvements than actual treatment allocation, analyses of covariance were conducted for treatment allocation with both longer-term treatments combined and with presumed antibiotic study medication per outcome measure (PCS, fatigue, MCS at both end-of-treatment and follow-up), including sex and baseline HRQoL as covariates in line with the analyses performed on the PLEASE trial [3]. Although the power analysis was based on the main research question of the PLEASE-trial [3], the smallest sample size of 170 patients indicated adequate power according to the rule of thumb of at least 10 participants per predictor variable [37]. All analyses were conducted with SPSS 25 and significance was accepted at *p* < 0.05.

## Results

### Descriptive statistics

The antibiotic treatment itself showed a very low drop-out, with 90% of randomized participants (252) completing the oral regimen of active study drug or placebo. In order to optimize comparability between analyses, for the current research questions, only those participants were included who had complete data with regard to all variables included in the study at either the end-of-treatment or the follow-up time point. Thus, this led to a total of 231 complete data sets at end-of treatment and 170 at follow-up (66 drop-outs between baseline and follow-up and 5 inclusions at follow-up only, because of missed outcome values at end-of-treatment).

Table 1 depicts the demographic, disease-related, and study-related characteristics, and baseline, end-of-treatment, and follow-up scores on the primary and secondary outcome measures, and baseline scores on the pre-treatment individual characteristics of the patients with complete end-of-treatment or follow-up data. The patients who did not complete all measures included at follow-up (*n* = 61) did not differ on most variables from the completer sample (*n* = 170), including all HRQoL measures, but were somewhat younger (*p* = 0.001) and had an overall lower acceptance (*p* = 0.04) and higher extraversion level (*p* = 0.001) than the completer sample. More specific information on the completer versus non-completer samples at both time points can be found in Appendix Tables 4 and 5.

**Table 1:** Descriptive statistics of the demographic, disease-related, study-related, outcome, and individual characteristics of the patients with complete data at end-of-treatment (14 weeks, *n* = 231) or follow-up (52 weeks, *n* = 170)

Variables	End-of-treatment sample (*n* = 231)	Follow-up sample (*n* = 170)
Demographic factors		
Age (mean (SD))	49.88 (11.70)	51.19 (11.55)
Sex (female) (*n* (%))	106 (45.9)	82 (48.2)
Steady partner (*n* (%))	201 (87.0)	149 (87.6)
Education level (*n* (%))		
Primary	1 (0.4)	1 (0.6)
Secondary	123 (53.2)	84 (49.4)
Tertiary	107 (46.3)	85 (50.0)
Smoking (*n* (%))	56 (24.2)	33 (19.4)
Paid labor (*n* (%))	143 (61.9)	102 (60.0)
Disease-related factors		
Duration of symptoms attributed to Lyme disease (years) (median (IQR))	2.47 (1.16–6.39)	2.26 (1.15–6.31)
Use of pain medication (*n* (%))	162 (70.1)	115 (67.6)
Study-related factors (*n* (%))		
Treatment arm		
Ceftriaxone followed by doxycycline	65 (28.1)	47 (27.6)
Ceftriaxone followed by clarithromycin and hydroxychloroquine	81 (35.1)	59 (34.7)
Ceftriaxone followed by placebo	85 (36.8)	64 (37.6)
Health-related quality of life (mean (SD))		
Physical HRQoL		
Physical component summary score (RAND SF-36, *T*-score)		
Pre-treatment	31.81 (7.47)	31.91 (7.50)
End-of-treatment (14 weeks)	36.21 (10.16)	36.14 (10.16)
Follow-up (52 weeks)		37.60 (11.38)
Fatigue severity (CIS)		
Pre-treatment	43.79 (10.09)	44.09 (9.71)
End-of-treatment (14 weeks)	37.17 (13.40)	37.07 (13.80)
Follow-up (52 weeks)		35.72 (14.66)
Mental HRQoL		
Mental component summary score (RAND SF-36, *T*-score)		
Pre-treatment	37.75 (9.57)	37.38 (9.36)
End-of-treatment (14 weeks)	41.42 (11.28)	41.51 (11.34)
Follow-up (52 weeks)		42.08 (11.60)
Individual characteristics (mean (SD))		
Expectancies regarding symptom improvement	16.12 (4.46)	16.25 (4.43)
Self-efficacy (LSE)	17.23 (5.36)	17.37 (4.99)
Illness cognitions (ICQ)		
Helplessness regarding disease	13.50 (4.24)	13.52 (4.34)
Disease acceptance	13.87 (3.89)	14.02 (3.90)
Perceived disease benefits	11.64 (4.13)	11.69 (4.21)
Worrying (PSWQ)	41.91 (12.28)	42.16 (12.31)
Personality (EPQ)		
Neuroticism	7.94 (5.16)	7.87 (5.24)
Extraversion	11.49 (4.63)	10.79 (4.50)

*CIS*, Checklist Individual Strength – Fatigue severity subscale; *EPQ*, Eysenck Personality Questionnaire; *ICQ*, Illness Cognition Questionnaire; *IQR*, interquartile range; *LSE*, Lyme Self-Efficacy; *PSWQ*, Penn-State Worry Questionnaire; *RAND SF-36*, RAND-36 Health Status Inventory; *SD*, standard deviation

Across groups, all quality of life outcome measures showed significant HRQoL improvements from pre-treatment to end-of-treatment (14 weeks; all *p*-values < 0.001), with further improvement (physical component summary score, *p* = 0.02) or stabilization of the improvement (fatigue, *p* = 0.09; mental component summary score, *p* = 0.46) at follow-up (52 weeks). Differentiating longer-term versus shorter-term treatment showed similar findings for fatigue and mental HRQoL, and a continued improvement vs. stabilization in the physical component summary score in the combined longer-term treatment arms vs. the shorter-term treatment arm (*p* = 0.045 vs. 0.19). In Appendix 1, the associations between the pre-treatment individual characteristics and demographic and disease-related factors are described.

### Pre-treatment predictors of quality of life course

Table 2 shows the main results of the hierarchical regression analyses in the total group examining the prediction of quality of life course after antimicrobial therapy based on those pre-treatment variables that correlated with the HRQoL at end-of-treatment (14 weeks) or follow-up (52 weeks). Having a partner, education level, duration of symptoms attributed to Lyme disease, helplessness, disease benefits, and extraversion did not significantly add to explaining the variance in any of the outcome measures. Therefore, to present the most parsimonious model, these variables were excluded from the final regression models.

**Table 2:** Percentage of explained variance and standardized regression coefficients (95% confidence intervals) of predictors of physical and mental health–related quality of life at end-of-treatment (14 weeks, *n* = 231) and follow-up (52 weeks, *n* = 170) in the total group (shorter- and longer-term treatment arms combined)

	Predictor	Physical HRQoL				Mental HRQoL	
		Physical component summary score (RAND-36 PCS)		Fatigue severity (CIS)		Mental component summary score (RAND-36 MCS)	
		Week 14	Week 52	Week 14	Week 52	Week 14	Week 52
Demographic characteristics	Δ*R*^2^	.12†	.08**	.07†	.07**	.05**	.08**
	Paid labor	.17* (1.56; 5.49)	.13* (.34; 5.87)	–.14** (–6.54; –1.19)	–.13* (–7.17; –.39)	.12* (.59; 4.91)	.16** (1.32; 6.34)
	Smoking	–.04 (–3.04; 1.24)	–.08 (–5.52; 1.15)	.02 (–2.24; 3.66)	.10# (–.45; 7.80)	–.09# (–4.63; .15)	–.12* (–6.70; − .57)
Pre-treatment HRQoL	Δ*R*^2^	.38†	.27†	.35†	.34†	.40†	.36†
	Pre-treatment PCS/CIS/MCS	.52† (.55; .86)	.36† (.32; .76)	.47† (.48; .78)	.47† (.52; .91)	.38† (.30; .60)	.38† (.30; .65)
Individual characteristics	Δ*R*^2^	.05†	.10†	.08†	.09†	.09†	.11†
	Expectancies symptom improvement	.14** (.09; .54)	.23† (.27; .92)	–.20† (–.89; –.30)	–.22† (–1.10; –.32)	.15** (.13; .61)	.22† (.27; .85)
	Self-efficacy (LSE)	.09 (–.04; .38)	.16* (.05; .69)	–.13* (–.61; –.04)	–.08 (–.62; .14)	.13* (.06; .50)	.13* (.02; .56)
	Worrying (PSWQ)	–.08 (–.17; .04)	.04 (–.12; .19)	.05 (–.09; .20)	.03 (–.16; .24)	–.17* (–.28; –.03)	–.06 (–.21; .10)
	Neuroticism (EPQ)	–.10 (–.45; .05)	–.17# (–.76; .004)	.12# (–.02; .66)	.14# (–.07; .87)	–.17* (–.65; –.01)	–.21* (–.82; –.10)
	Total *R*^2^	.54†	.45†	.50†	.50†	.54†	.56†

#*p* < .10, * *p* < .05, ** *p* < .01, † *p* < .001; percentage of explained variance (Δ and total *R*^2^), and standardized regression coefficients (*β*, (95% CI)) were assessed by means of hierarchical regression analyses; predictors were included when at least one significant association was found in prior regression analyses including all predictors showing any significant zero-order association with any of the outcome measures at end-of-treatment (14 weeks) or follow-up (52 weeks)

Abbreviations: *CIS*, Checklist Individual Strength - Fatigue severity subscale; *EPQ*, Eysenck Personality Questionnaire; *HRQoL*, health-related quality of life; *ICQ*, Illness Cognition Questionnaire; *LSE*, Lyme Self-Efficacy scale; *RAND-36*, RAND SF-36 Health Status Inventory - MCS, mental component summary score; *PSWQ*, Penn-State Worry Questionnaire; *RAND-36 PCS*, RAND SF-36 Health Status Inventory – physical component summary score

For all physical and mental HRQoL measures, each separate block of pre-treatment demographic characteristics, pre-treatment functioning, and pre-treatment individual characteristics significantly added explained variance to the model, with a total explained variance between 40 and 56%, depending on the outcome measure. The largest amount of variance (27–40%) was explained by pre-treatment functioning on that particular outcome measure. The demographic characteristics paid labor (predicting better physical and mental HRQoL) and smoking (predicting worse mental HRQoL, mainly at follow-up) added 5 to 12%. The pre-treatment individual characteristics added 5 to 11% to the explained variance (Table 2).

In the total group, pre-treatment expectancies regarding symptom improvement consistently predicted physical and mental HRQoL at end-of-treatment on top of pre-treatment functioning, thus predicting actual HRQoL improvement. This effect was even stronger at 1 year after the start of treatment (follow-up). Less consistently than pre-treatment expectancies, the other individual characteristics predicted mental (higher self-efficacy, less worrying, and lower neuroticism) and physical (higher self-efficacy) HRQoL improvement (Table 2).

Sensitivity analyses for shorter-term and longer-term treatment arms are reported in Table 3. Stratification of the duration of antimicrobial treatment (shorter-term versus longer-term treatment) overall showed a similar pattern of associations, with pre-treatment expectancies being the most consistent predictor of HRQoL at end-of-treatment and follow-up in both groups (Table 3). Differences between treatment arms consisted of the lack of predictive value of the demographic characteristics in the shorter-term antimicrobial treatment arm as opposed to the longer-term treatment arms (1–4% vs. 9–18% explained variance) and the larger predictive value of pre-treatment mental HRQoL for end-of-treatment and follow-up mental HRQoL in the shorter-term than longer-term treatment arms (51–52% vs. 28–34% explained variance).

**Table 3:** Percentage of explained variance and standardized regression coefficients (95% confidence intervals) of predictors of physical and mental health–related quality of life at end-of-treatment (14 weeks, *n* = 231) and follow-up (52 weeks, *n* = 170) in the shorter-term (ST) versus longer-term (LT) treatment arms

	Predictor variable	Rx	Physical HRQoL				Mental HRQoL	
			Physical component summary score (RAND-36 PCS)		Fatigue severity (CIS)		Mental component summary score RAND-36 (MCS)	
			Week 14	Week 52	Week 14	Week 52	Week 14	Week 52
Demographic characteristics	Δ*R*^2^	ST LT	.04 .18†	.04 .14**	.02 .13†	.01 .14†	.01 .09**	.01 .16†
	Paid labor	ST LT	.09 (–1.33; 5.32) .23† (2.08; 7.22)	.16 (–1.47; 9.50) .11 (–1.04; 5.70)	–.08 (–7.08; 2.45) –.17** (–8.01; –1.21)	–.08 (–8.91; 3.95) –.14* (–8.20; –.02)	.04 (–2.62; 4.56) .16** (.99; 6.59)	.07 (–2.73; 6.36) .19** (1.08; 7.44)
	Smoking	ST LT	–.04 (–4.52; 2.82) –.04 (–3.82; 1.82)	–.04 (–7.51; 5.27) –.12 (–7.28; .76)	–.05 (–6.67; 3.80) .04 (–2.52; 5.08)	.03 (–6.55; 8.61) –.14* (–8.20; –.02)	–.06(–5.62; 2.33) –.07 (–4.90; 1.37)	–.01 (–5.61; 5.27) –.17* (–8.62; − .94)
Pre-treatment HRQoL	Δ*R*^2^	ST LT	.51† .30†	.27† .26†	.39† .32†	.36† .31†	.53† .34†	.51† .28†
	Pre-treatment PCS/CIS/MCS	ST LT	.58† (.49; .99) .48† (.47; .89)	.28# (–.01; .86) .39† (.33; .84)	.50† (.35; .90) .46† (.45; .82)	.44** (.30; 1.08) .49† (.50; .97)	.60† (.43; .94) .29† (.16; .53)	.64† (.50; 1.12) .26** (.11; .53)
Individual characteristics	Δ*R*^2^	ST LT	.04 .05**	.11* .11**	.09* .08†	.11* .09**	.05# .12†	.09* .13†
	Expectancies symptom improvement	ST LT	.12 (–.10; .58) .15* (.06; .66)	.18 (–.12; 1.01) .30† (.38; 1.24)	–.18* (–.97; –.01) –.20** (–1.05; –.25)	–.18# (–1.20; .09) –.28† (–1.43; –.46)	.13* (–.07; .66) .15* (.08; .74)	.26** (.17; 1.09) .22** (.20; 1.01)
	Self-efficacy (LSE)	ST LT	.12 (–.13; .54) .07 (–.16; .42)	.19 (–.17; 1.05) .13 (–.09; .68)	–.07 (–.66; .32) –.15* (–.78; –.03)	–.07 (–.91; .52) –.05 (–.59; .29)	.004 (–.34; .36) .19** (.12; .71)	–.04 (–.58; .40) .18* (.09; .75)
	Worrying (PSWQ)	ST LT	–.08 (–.25; .11) –.08 (–.20; .07)	–.08 (–.40; .23) .17 (–.06; .36)	.05 (–.21; .33) .05 (–.13; .24)	.18 (–.15; .60) .10 (–.10; .33)	–.04 (–.24; .17) –.24** (–.38; –.06)	–.001 (–.27; .26) –.07 (–.27; .14)
	Neuroticism (EPQ)	ST LT	–.08 (–.58; .25) –.11 (–.54; .12)	–.16 (–1.09; .33) –.25* (–.99; –.03)	.18 (–.13; 1.07) .09 (–.21; .67)	.11 (–.52; 1.17) .03 (–.44; .63)	–.18# (–.87; .08) –.14# (–.68; .06)	–.13 (–.94; .32) –.25* (–1.00; − .06)
	Total *R*^2^	ST LT	.59† .53†	.41† .51†	.49† .53†	.47† .55†	.59† .55†	.60† .57†

#*p* < .10, *p < .05, **p < .01, †*p* < .001; percentage of explained variance (Δ and total *R*^2^) and standardized regression coefficients (*β*, (95% CI)) were assessed by means of hierarchical regression analyses; predictors were included in case at least one significant association was found in prior regression analyses including all predictors showing any significant zero-order association with any of the outcome measures at end-of-treatment (14 weeks) or follow-up (52 weeks)

Abbreviations: *CIS*, Checklist Individual Strength - Fatigue severity subscale; *EPQ*, Eysenck Personality Questionnaire; *HRQoL*, health-related quality of life; *ICQ*, Illness Cognition Questionnaire; *LSE*, Lyme Self-Efficacy scale; *RAND-36*, RAND SF-36 Health Status Inventory - MCS, mental component summary score; *PSWQ*, Penn-State Worry Questionnaire; *RAND-36 PCS*, RAND SF-36 Health Status Inventory – physical component summary score; *Rx*, shorter-term (ST) or longer-term (LT) antimicrobial therapy

### Post-treatment expectancies of presumed study medication being associated with quality of life course

At end-of-treatment, more patients presumed to have received antibiotics (*n* = 148, 64.1%) than placebo (*n* = 32, 13.9%) during blinded randomized treatment; the remainder indicated not to know (*n* = 51, 22.1%). A significant difference in presumed study medication was found between the treatment arms, with a larger percentage of patients in the longer-term antibiotics group who (adequately) presumed to have received antibiotics (71.2%) compared to the placebo group (51.8%; *p* = 0.007), with no difference between the two longer-term groups (doxycycline: 63.1%; clarithromycin: 77.8%; *p* = 0.12).

When post-treatment expectancies of presumed study medication were included as a post-treatment predictor in the hierarchical regression analyses in an additional block, the explained variance of quality of life at end-of-treatment significantly increased by 2 to 3%, with patients who thought to have received antibiotics showing improvements in all outcome measures at end-of-treatment (14 weeks; PCS: *β* = 0.14, *p* = 0.003, 95% CI 1.00;4.75; CIS: *β* =  − 0.17, *p* < 0.001, 95% CI −7.34;−2.22; MCS: *β* = 0.17, *p* < 0.001, 95% CI 1.95;6.07). Post-treatment expectancies did not add significantly to physical or mental HRQoL at follow-up (52 weeks; PCS: *p* = 0.22; CIS: *p* = 0.24; MCS: *p* = 0.07). These effects were similar in the shorter-term and longer-term treatment arms for end-of-treatment (14 weeks), whereas post-treatment expectancies were significantly (or with a trend towards significance) associated with physical and mental HRQoL at follow-up in the longer-term treatment arms only (PCS: *p* = 0.86 vs. 0.08; CIS: *p* = 0.78 vs. 0.03; MCS: *p* = 0.70 vs. 0.03 in the shorter-term vs. longer-term treatment arms).

In the previous PLEASE trial [3], it was shown that the antibiotics treatments on top of the short-term ceftriaxone treatment did not lead to a different change in physical or mental HRQoL at end-of-treatment and follow-up than when short-term treatment was followed by placebo. Within the current study, this finding was confirmed when combining the two longer-term antibiotics treatments, showing no significant effect of group on physical or mental HRQoL, when controlling for gender and HRQoL at baseline (PCS: 14 weeks: *p* = 0.26; 52 weeks: *p* = 0.77; CIS: 14 weeks: *p* = 0.93; 52 weeks: *p* = 0.40; MCS: 14 weeks: *p* = 0.43; 52 weeks: *p* = 0.63). Entering whether participants presumed to have received antibiotics instead of actual group allocation showed significant effects on all measures of HRQoL at end-of-treatment (14 weeks; PCS: *F*(1,230) = 9.78, *p* = 0.002; CIS: *F*(1,230) = 12.59, *p* < 0.001; MCS: *F*(1,230) = 12.94, *p* < 0.001). For all HRQoL measures, quality of life was reported to be better in the group of participants who presumed to have received antibiotics (presumed vs. non-presumed antibiotics received: PCS: 37.44 ± 10.23 vs. 34.02 ± 9.72; CIS: 35.39 ± 13.61 vs. 40.33 ± 12.48; MCS: 42.88 ± 11.31 vs. 38.82 ± 10.80). These effects were no longer significant at follow-up (52 weeks; PCS: *F*(1,169) = 2.22, *p* = 0.14; CIS: *F*(1169) = 1.85, *p* = 0.18; *F*(1,169) = 3.70, *p* = 0.06).

## Discussion

The current study examined the role of expectancies regarding symptom improvement and other individual characteristics in predicting quality of life course after antimicrobial therapy for persistent symptoms attributed to Lyme disease. In addition to pre-treatment functioning, pre-treatment expectancies regarding symptom improvement and post-treatment expectancies on having received antibiotics were found to be consistent predictors of larger improvements in physical and mental HRQoL. Other individual characteristics, related to more generalized outcome expectancies, showed less consistent predictive associations. Thus, where the main outcomes of our PLEASE-trial showed that the two specific standardized longer-term antimicrobial therapies did not have significant added benefits over shorter-term treatment for the quality of life outcomes at group level [3], the current study showed that expectancies regarding symptom improvement and received study medication are associated with symptom course after both shorter-term and longer-term antimicrobial treatment. These findings suggest that expectancies are relevant to consider in treatment studies and may be useful in clinical settings to improve symptom course and treatment outcome, which is in line with the current upsurge of research into the clinical potential of optimization of placebo effects and minimization of nocebo effects [23, 27, 38, 39].

The role of positive or negative expectancies, for example regarding symptom course or treatment outcome, has been studied mostly within the area of placebo research. In this research, it has for instance been shown that induction of positive expectancies by means of learning procedures such as conditioning and verbal suggestions leads to decreased experience of physical symptoms such as pain and itch [40, 41]. Placebo effects have traditionally mainly been examined in the context of placebo-controlled randomized trials to discriminate the “real” treatment effect from other “random” effects [42]. Currently, however, evidence has been accumulating that indicates the large clinical potential of implementing the placebo effect into the clinic to optimize patient care [23]. Situation-specific or treatment-specific expectancies have not often been examined as predictors of treatment outcome or symptom course in clinical trials up to now. This is in contrast to more generalized outcome expectancy characteristics, such as the tendency to have faith in one’s abilities to deal with adversities (i.e., self-efficacy), to worry about potential negative future events (i.e., worrying), and to experience negative emotional states and to view the world as threatening (i.e., neuroticism). The current study examined the relative predictive contribution of situation-specific expectancies next to other potentially relevant individual characteristics, of which only these more generalized outcome expectancies were found to be relevant. The results clearly showed that situation-specific pre-treatment and post-treatment expectancies were the most consistent predictors of quality of life course at end-of-treatment and, for pre-treatment expectancies, even more strongly at follow-up. Of the more generalized outcome expectancy characteristics, less consistent associations were found for self-efficacy, worrying, and neuroticism. Although in line with previous studies in other chronic conditions [9, 11–13], it indicates less consistent evidence regarding the potential predictive value of more generalized as opposed to situation-specific outcome expectancies. This agrees with the findings of our previous study on an immune-related training program in healthy men, in which situation-specific outcome expectancies were found to be associated to clinical symptom report after endotoxin administration [18]. The findings of the current study thus suggest the added value of both pre-treatment and post-treatment expectancies in explaining individual differences in treatment success regarding symptom course or treatment outcome in patients with persistent symptoms attributed to Lyme disease and possibly also other chronic conditions.

Patients with persistent symptoms attributed to Lyme disease report a high symptom burden and disability, and low quality of life [1, 3]. As specific standardized protocols of prolonged antimicrobial therapy have mostly not lead to improved treatment outcomes at group level [3–5], it is relevant to find factors associated with outcomes that treatment could be tailored to or other ways to improve symptom course and treatment outcome for this patient group. Patients with persistent symptoms for whom there is no gold standard treatment, such as the patients in our study, will have a high chance of having been confronted with negative treatment experiences. These negative experiences will automatically and unintentionally lead to negative outcome expectancies regarding new treatments. To prevent further disappointment, health care professionals tend to be hesitant to induce any positive expectancies in their patients [43]. However, as the current study illustrates, pre-treatment expectancies of symptom improvement are relevant predictors of quality of life in both the shorter-term and longer-term treatment arms. This underscores the relevance of examining different ways to optimize expectancies in clinical practice to potentially improve treatment outcomes in this high-burdened patient group, for example by means of enhanced doctor-patient communication and open-label placebo treatments [44] in which patients are informed about receiving a placebo and its working mechanisms. That the most consistent and long-lasting effects were found for the patients receiving longer-term antimicrobial therapy, specifically regarding post-treatment expectancies, may reflect that patients did notice somehow whether or not they received longer-term antibiotics, which probably has impacted their expectancies. Alternatively, it could be explained by the lower power in the shorter-term group, as the two longer-term treatment arms were combined in the analyses.

The current study extended on the main findings of the PLEASE trial by showing that the improvements in quality of life from pre-treatment up to 1 year after start of treatment, which could on group level not be attributed to added benefits of the specific longer-term antimicrobial treatment regimens provided on top of 2-week ceftriaxone treatment [3], are associated with pre-treatment expectancies regarding symptom improvement. Also, the current findings suggest treatment outcome to be more strongly associated with presumed antibiotic use than with actual antibiotic use. The fact that the question on presumed medication use was merely asked immediately after treatment allows for a bidirectional interpretation of the findings (i.e., treatment improvements impacting on the belief that one has received antibiotics versus believing one has received antibiotics impacting on treatment outcomes). However, presumed medication use remained a significant, although less strong, predictor of outcomes up to 1 year after start of treatment (38 weeks after presumed medication assessment) within the longer-term treatment arms. Thus, although the two types of longer-term antimicrobial therapy have not shown at group level to be more effective than the 2-week antimicrobial treatment that all patients in this study received, our patients may ascribe positive expectancies towards this treatment, which are related to a more positive outcome. This suggests the relevance of optimizing patient expectancies before the start of new treatment, of course within ethical boundaries [27].

Strengths of the current study include the large sample size and rigorous RCT study design. Also, the inclusion of pre-treatment functioning in the regression analysis provides a more stringent test of the added value of individual characteristics in actually predicting the change in HRQoL from baseline to end-of-treatment or follow-up. Limitations include the self-report nature of all predictor and outcome measures, allowing potential response bias effects. Also, the difference in patient numbers at end-of-treatment and follow-up prevents direct comparability of findings, and the lower power in the shorter-term compared to the combined longer-term treatment arms complicates the interpretation of differences in predictions between groups. Finally, the assessment of post-treatment expectancies brings inherent interpretability problems due to its assessment being intertwined with outcome assessment.

To conclude, the present study shows how patients’ pre-treatment and post-treatment expectancies regarding improvement of persistent symptoms attributed to Lyme disease can, next to pre-treatment functioning, explain a more beneficial symptom course. It would be relevant to examine in future research how expectancies could be optimized in patients with persistent symptoms attributed to Lyme disease, for instance by enhanced doctor-patient communication, in order to potentially improve symptom course and treatment effectiveness. These results underscore recommendations to (1) ascertain that patient pre-treatment expectancies are realistic and can be met, and (2) inform patients and clinicians about the role of expectancies and taking these into account in treatments and research trials.

## Data Availability

The datasets generated during and/or analyzed during the current study are available from the corresponding author on reasonable request.

## Appendix

**Table 4:** Descriptive statistics and comparisons of the demographic, disease-related, study-related, outcome, and individual characteristics of the completer sample at end-of-treatment (14 weeks; *n* = 231) versus non-completers (*n* = 49)

Variables	Completer end-of-treatment sample (*n* = 231)	Non-completer end-of-treatment sample (*n* = 49)	*p*-value
Demographic factors			
Age (mean (SD))	49.88 (11.70)	46.42 (12.44)	0.06
Sex (female) (*n* (%))	106 (45.9)	23 (46.9)	0.89
Steady partner (*n* (%))	201 (87.0)	38 (80.9)	0.27
Education level (*n* (%))			0.31
Primary	1 (0.4)	0 (0.0)	
Secondary	123 (53.2)	30 (65.2)	
Tertiary	107 (46.3)	16 (34.8)	
Smoking (*n* (%))	56 (24.2)	8 (16.7)	0.26
Paid labor (*n* (%))	143 (61.9)	28 (59.6)	0.77
Disease-related factors			
Duration of symptoms attributed to Lyme disease (years) (median (IQR))	2.47 (1.16–6.39)	2.55 (1.27–4.27)	0.24
Use of pain medication (*n* (%))	162 (70.1)	28 (59.6)	0.16
Study-related factors (*n* (%))			
Treatment arm			0.12
Ceftriaxone followed by doxycycline	65 (28.1)	21 (42.9)	
Ceftriaxone followed by clarithromycin and hydroxychloroquine	81 (35.1)	15 (30.6)	
Ceftriaxone followed by placebo	85 (36.8)	13 (26.5)	
Health-related quality of life (mean (SD))			
Physical HRQoL			
Physical component summary score (RAND SF-36, *T*-score)			
Pre-treatment	31.81 (7.47)	30.90 (7.13)	0.44
End-of-treatment (14 weeks)	36.21 (10.16)	38.50 (11.19; *n* = 30)	0.25
Fatigue severity (CIS)			
Pre-treatment	43.79 (10.09)	45.62 (9.48)	0.25
End-of-treatment (14 weeks)	37.17 (13.40)	34.92 (15.09; *n* = 31)	0.39
Mental HRQoL			
Mental component summary score (RAND SF-36, *T*-score)			
Pre-treatment	37.75 (9.57)	35.49 (10.44)	0.15
End-of-treatment (14 weeks)	41.42 (11.28)	45.81 (13.16; *n* = 26)	0.11
Individual characteristics (mean (SD))			
Expectancies regarding symptom improvement	16.12 (4.46)	17.49 (4.02)	0.07
Self-efficacy (LSE)	17.23 (5.36)	16.21 (5.13)	0.23
Illness cognitions (ICQ)			
Helplessness regarding disease	13.50 (4.24)	13.94 (4.52)	0.52
Disease acceptance	13.87 (3.89)	12.53 (4.11)	0.03
Perceived disease benefits	11.64 (4.13)	10.93 (3.93)	0.28
Worrying (PSWQ)	41.91 (12.28)	43.39 (13.48)	0.46
Personality (EPQ)			
Neuroticism	7.94 (5.16)	9.51 (5.36)	0.06
Extraversion	11.49 (4.63)	11.89 (5.22)	0.62
Presumed medication use (*n* (%))			0.52
Antibiotics	148 (64.1)	26 (72.2)	
Placebo	32 (13.9)	5 (13.9)	
Not known	51 (22.1)	5 (13.9)	

*CIS*, Checklist Individual Strength – Fatigue Severity Subscale; *EPQ*, Eysenck Personality Questionnaire; *ICQ*, Illness Cognition Questionnaire; *IQR*, interquartile range; *LSE*, Lyme Self-Efficacy; *PSWQ*, Penn-State Worry Questionnaire; *RAND SF-36*, RAND-36 Health Status Inventory; *SD*, standard deviation

**Table 5:** Descriptive statistics and comparisons of the demographic, disease-related, study-related, outcome, and individual characteristics of the completer sample at follow-up (52 weeks; *n* = 170) versus drop-outs between end-of-treatment (14 weeks) and follow-up (52 weeks; *n* = 66)

Variables	Completer follow-up sample (*n* = 170)	Drop-outs between end-of-treatment and follow-up (*n* = 66)	*p*-value
Demographic factors			
Age (mean (SD))	51.19 (11.55)	45.88 (11.85)	0.002
Sex (female) (*n* (%))	82 (48.2)	26 (39.4)	0.22
Steady partner (*n* (%))	149 (87.6)	57 (86.4)	0.79
Education level (*n* (%))			0.13
Primary	1 (0.6)	0 (0.0)	
Secondary	84 (49.4)	42 (63.6)	
Tertiary	85 (50.0)	24 (36.4)	
Smoking (*n* (%))	33 (19.4)	23 (34.8)	0.01
Paid labor (*n* (%))	102 (60.0)	44 (66.7)	0.34
Disease-related factors			
Duration of symptoms attributed to Lyme disease (years) (median (IQR))	2.26 (1.15–6.31)	2.75 (1.20–6.42)	0.56
Use of pain medication (*n* (%))	115 (67.6)	50 (75.8)	0.22
Study-related factors (*n* (%))			
Treatment arm			0.82
Ceftriaxone followed by doxycycline	47 (27.6)	20 (30.3)	
Ceftriaxone followed by clarithromycin and hydroxychloroquine	59 (34.7)	24 (36.4)	
Ceftriaxone followed by placebo	64 (37.6)	22 (33.3)	
Health-related quality of life (mean (SD))			
Physical HRQoL			
Physical component summary score (RAND SF-36, *T*-score)			
Pre-treatment	31.91 (7.50)	31.48 (7.32)	0.70
End-of-treatment (14 weeks)	36.14 (10.16)	36.58 (10.28)	0.77
Fatigue severity (CIS)			
Pre-treatment	44.09 (9.71)	43.16 (10.85)	0.52
End-of-treatment (14 weeks)	37.07 (13.80)	37.24 (12.29)	0.93
Mental HRQoL			
Mental component summary score (RAND SF-36, *T*-score)			
Pre-treatment	37.38 (9.36)	38.47 (9.95)	0.43
End-of-treatment (14 weeks)	41.51 (11.34)	41.44 (11.31)	0.97
Individual characteristics (mean (SD))			
Expectancies regarding symptom improvement	16.25 (4.43)	15.83 (4.44)	0.51
Self-efficacy (LSE)	17.37 (4.99)	16.83 (6.20)	0.53
Illness cognitions (ICQ)			
Helplessness regarding disease	13.52 (4.34)	13.48 (4.01)	0.96
Disease acceptance	14.02 (3.90)	13.43 (3.83)	0.29
Perceived disease benefits	11.69 (4.21)	11.44 (3.94)	0.67
Worrying (PSWQ)	42.16 (12.31)	41.60 (12.40)	0.76
Personality (EPQ)			
Neuroticism	7.87 (5.24)	8.17 (4.96)	0.69
Extraversion	10.79 (4.50)	13.51 (4.43)	< 0.001
Presumed medication use (*n* (%))			0.87
Antibiotics	111 (65.3)	41 (62.1)	
Placebo	23 (13.5)	9 (13.6)	
Not known	36 (21.2)	16 (24.2)	

*CIS*, Checklist Individual Strength – Fatigue severity subscale; *EPQ*, Eysenck Personality Questionnaire; *ICQ*, Illness Cognition Questionnaire; *IQR*, interquartile range; *LSE*, Lyme Self-Efficacy; *PSWQ*, Penn-State Worry Questionnaire; *RAND SF-36*, RAND-36 Health Status Inventory; *SD*, standard deviation
